# Hematopoietic Stem-Cell Transplantation in the Developing World: Experience from a Center in Western India

**DOI:** 10.1155/2015/710543

**Published:** 2015-02-03

**Authors:** Chirag A. Shah, Arun Karanwal, Maharshi Desai, Munjal Pandya, Ravish Shah, Rutvij Shah

**Affiliations:** Apollo Hospitals International Limited, Plot No. 1 A, Bhat GIDC Estate, Gandhinagar, Gujarat 382428, India

## Abstract

We describe our experience of first 50 consecutive hematopoietic stem-cell transplants (HSCT) done between 2007 and 2012 at the Apollo Hospital, Gandhinagar, 35 autologous HSCT and 15 allogeneic HSCT. Indications for autologous transplant were multiple myeloma, non-Hodgkin lymphoma, Hodgkin lymphoma, and acute myeloid leukemia, and indications for allogeneic transplants were thalassemia major, aplastic anaemia, chronic myeloid leukemia, and acute lymphoblastic and myeloid leukaemia. The median age of autologous and allogeneic patient's cohort was 50 years and 21 years, respectively. Median follow-up period for all patients was 39 months. Major early complications were infections, mucositis, acute graft versus host disease, and venoocclusive disease. All of our allogeneic and autologous transplant patients survived during the first month of transplant. Transplant related mortality (TRM) was 20% (*N* = 3) in our allogeneic and 3% (*N* = 1) in autologous patients. Causes of these deaths were disease relapse, sepsis, hemorrhagic complications, and GVHD. 46% of our autologous and 47% of our allogeneic patients are in complete remission phase after a median follow-up of 39 months. 34% of our autologous patients and 13% of our allogeneic patients had disease relapse. Overall survival rate in our autologous and allogeneic patients is 65.7% and 57.1%, respectively. Our results are comparable to many national and international published reports.

## 1. Introduction

Trends of hematopoietic stem-cell transplantation (HSCT) evolved with the first successful transplantation done by Dr. E. Donnall Thomas in late 1950s, for which he received the Nobel Prize in Physiology or Medicine in 1990. That transplant was done between the identical twins in a case of leukemia [1]. In 1968, in Minnesota, the first successful nontwin (allogeneic) transplant was performed. In this case, the donor was a sibling of the patient. By this time, it was known that a key to a successful transplant was a specific type of genetic matching (known as HLA) of the donor to the patient [1]. The first successful unrelated donor transplant was done in 1973, when a young kid in New York with acute leukemia received multiple bone marrow transplants from a matched donor from Denmark [1]. The application of hematopoietic stem-cell transplantation is not new in India. India's first successful allogeneic bone marrow transplantation was done at Tata Memorial Hospital on March 20, 1983, on a nine-year-old girl with acute myeloid leukemia [2]. Since then, many sophisticated hematopoietic stem-cell transplant centers (HSCT) have been established across the nation. Until September 2005, data from six transplant centres in India were collected and a total of 1540 transplants have been performed in a country of over one billion population [2]. In India, there are 11 centres currently reporting their data to CIBMTR (Center for International Blood and Marrow Transplant Research) [3]. However these numbers are not large, and centres which perform regular HSCT are low due to various reasons like lack of infrastructure and expertise and lack of knowledge of safety, efficacy, and cost of the procedure both in general population and in medical fraternity. Family genotype analysis in India reveals that 39.3% of the total numbers of patients have an HLA-matched sibling and that families with sibship size of more than or equal to 4 have a higher probability (68.8%) compared with those with sibship size of less than 4 (29.7%) [4]. Recently most of the transplant centres have started marrow unrelated donor (MUD) transplants and a few centres have started doing haploidentical transplants. Apollo Hospital International Limited, Gandhinagar, is one of the registered institutes of CIBMTR. Apollo CBCC comprehensive cancer center is located in the capital city of Gujarat, Gandhinagar, and it is one of the largest and busiest private hospitals of Western India. It also is one of the few bone marrow transplant centers in the state of Gujarat, which has population of about 60 millions [5].

*Description of Our Transplant Center.* Apollo Hospital, Gandhinagar, is an NABH accredited hospital, part of India's largest and most reputed healthcare groups. Apollo CBCC Comprehensive Cancer Center is the first private comprehensive cancer center of Gujarat state, established in 2007, in collaboration with CBCC (Comprehensive Blood and Caner Center), USA. Our hematooncology ward consists of 14 beds, which includes 6 rooms with HEPA filters (neutropenic ward). Our transplant unit consists of 6 beds and is totally isolated from the other part of the hospital with restricted entry of personnel and very strict neutropenic precautions including HEPA filtered air and positive pressure ventilation. Our transplant team consists of a transplant physician (hematooncologist), adult intensivists, pediatric and neonatal intensivists, oncologists, an infectious disease specialist, a registrar, medical officers, skilled nursing staff, neutropenic dieticians, well-trained nursing assistants, and managing staff, which is a perfect example of a multidisciplinary team efforts.

## 2. Baseline Data

### 2.1. Materials and Methods

This is a retrospective analysis of first fifty consecutive patients who undergone transplant at our institute, 15 allogeneic and 35 autologous with M : F = 41 : 9, whom have been followed at least for 22 months to measure their parameters accurately with median follow-up of 39 months with range from 22 to 82 months. The data was obtained carefully from case sheets, medical records of the hospital, and statistically analyzed. We receive patients of all diseases in which bone marrow transplant may be necessary and/or indicated. Our majority of pool is from western and central states of India including Gujarat, Rajasthan, Maharashtra, Madhya Pradesh, and some other states too. We receive international patients from Africa, Asia, and Middle East countries. Baseline patients' characteristics are shown in Table 1.

### 2.2. Trends in Transplants by Type and Recipient Age

**Figure 1:**
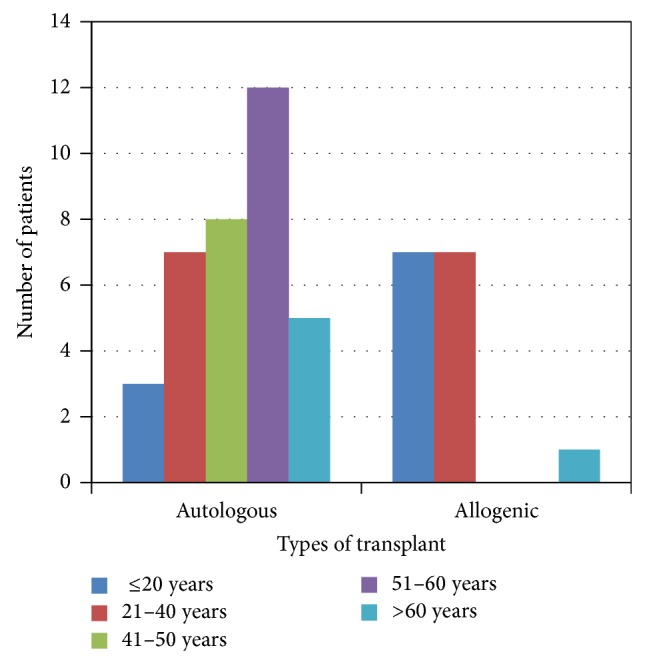
Age-wise distribution of transplant patients.

**Figure 2:**
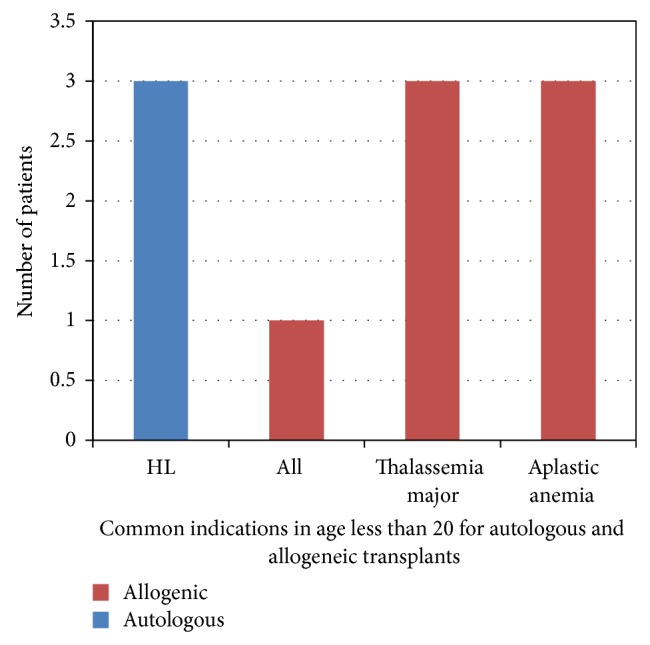
Major indications of transplant less than 20 years of age.

The median age of autologous BMT was 50 years and allogeneic BMT was 21 years. Majority of autologous transplants were done between the ages of 51 and 60 years and in patients with multiple myeloma, non-Hodgkin lymphoma, and Hodgkin lymphoma, which are more common in this age group. Major indications of allogeneic BMT in age group less than 20 years were thalassemia and aplastic anemia and for autologous BMT they were Hodgkin's lymphoma. Figures 1 and 2 show graphical presentation of age of distribution and major indications below age of 20.

### 2.3. Indications for Hematopoietic Stem-Cell Transplants

Common indications for autologous BMT were multiple myeloma (57%), non-Hodgkin's lymphoma (23%). Allogeneic BMTs were done in patients with acute lymphoblastic lymphoma relapse, chronic myeloid leukemia with imatinib failure, aplastic anemia, thalassemia, and myelodysplastic syndrome. Two AML relapse cases undergone allogeneic BMT whereas three AML patients undergone autologous BMT after induction and consolidation chemotherapy.

We started hematopoietic stem-cell transplantation in 2007. From the year 2007 till June 2012, numbers of transplants have gradually increased and in total we have completed 50 transplants.

### 2.4. Stem-Cell Collection

Stem cells were collected from peripheral blood in 92% [*n* = 46] of patients while in 8% [*n* = 4] patients bone marrow was the source. Till the date of analysis, we have not done allogeneic HSCT with stem-cells from cord blood, haploidentical donor, or marrow unrelated donor (MUD). All of the autologous stem-cells were collected from peripheral blood. All of our Allogeneic transplant patients were 6/6 HLA antigens matched according to standard protocols [12] except one patient in which we did transplant with 4/6 matched HLA antigens from sibling. Stem-cells from peripheral blood were collected after mobilization therapy consisted of G-CSF stimulation in the dose of 10 mcg/kg/day for 4-5 days and from bone marrow collected by standard methods. Median stem-cell dose (CD34 cells/kg) transplanted for our allogeneic BMT is 5.15^∧^10^6^ and 2.56^∧^10^6^ for autologous transplant patients. Autologous HSC were cryopreserved in a preservative called DMSO and the cells were cooled very slowly in a controlled rate freezer to avoid cell loss by osmotic injury [13]. Cryopreservation is necessary especially in autologous transplants because HSC collections are usually done much in advance of its transplant. We preferred allogeneic HSC collection to be done very near to the transplant to avoid cell loss during freezing and thawing.

### 2.5. Transplant Conditioning Protocols

All patients were given myeloablative conditioning chemotherapy without the use of total body irradiation (TBI). We have not used nonmyeloablative or reduced intensity regimens. Chemotherapy was given through central lines in all patients. BEAM regimen was used for Hodgkin and non-Hodgkin lymphomas. BuCy regimen was used for acute leukaemias. Melphalan was used for myelomas according to standard protocols [14].  Table 2 describes our major conditioning regimens.

### 2.6. Antimicrobial Prophylaxis

See Tables 4 and 5.

### 2.7. VOD Prophylaxis

This included tablets of Ursodeoxycholic acid 300 mg thrice daily to all allogeneic patients.

### 2.8. Graft versus Host Prophylaxis

Table 3 shows GVHD prophylaxis in our allogeneic HSCT patients.

## 3. Outcome Data

### 3.1. Posttransplant Complications: Early Complications within 100 Days after Transplant

The most common early posttransplant complications were mucositis, infections, venoocclusive disease (VOD), graft versus host disease (GVHD), and hemorrhagic cystitis. We have taken references from CTCAE (Common Terminology Criteria for Adverse Events) to grade our posttransplant complications [15].  Table 6 shows major early posttransplant complications in our autologous and allogeneic patients.

#### 3.1.1. Oral Mucositis

Oral mucositis was one of the most common complications of both allogeneic and autologous BMT. Four (27%) (two grade 2 and two grade 3) of our allogeneic transplant patients and five (15%) (one grade 1, two grade 2, and two grade 3) of our autologous transplant patients had mucositis [16]. Grade 2 mucositis patients were managed with dietary modification, analgesics, and oral care and patients with grade 3 required some kind of intervention like parental nutrition and/or feeding tube insertion. We use ice sucking during melphalan and fludarabine conditioning chemotherapy to prevent/reduce mucositis. Chemotherapy and HSCT were the probable causes of the mucositis [17].

#### 3.1.2. Infections

*Allogeneic BMT.* There were 22 febrile neutropenia incidences during the transplantation hospital stay, out of which in 17 (77%) infection was documented with culture positivity and 5 (23%) were culture negative. Major infections were bacterial followed by fungal and viral causes. Major bacteria were* Staphylococcus* (25%),* Pseudomonas* (20%), and* Bacillus* species (20%) and minor infections were* Streptococcus* (18%) and* E. coli*. Three incidences were fungal infections.* Candida* (66%) was the commonest cause followed by* Aspergillus* (33%). One* Cytomegalovirus* infection with significant viral copies was also noted which required Ganciclovir. Major sites of culture positivity were blood (40%) and respiratory tract (30%) and other were urine, stool, and CVP line.

*Autologous BMT.* There were 27 incidences of febrile neutropenia, out of which in 20 (74%) infection was documented with culture positivity. Major infections were bacterial followed by fungal and viral causes. Major bacterial infections were* Streptococcus* (25%),* E. coli* (22%),* S. aureus* (18%), and* Klebsiella* (18%) followed by other species like* Proteus*,* Pseudomonas*, and* Typhoid*. Major fungal infections were* Candida* (50%) followed by* Aspergillus* (25%),* Rhizopus*, and* Mucormycosis*. One incidence of* Cytomegalovirus* was noted with significant viral copies that required Ganciclovir and one incidence of Herpes zoster was noted. Major sites of culture positivity were respiratory tract (33%), blood (25%), and stool (25%) followed by urine, CVP/catheter tip, and wound.

#### 3.1.3. Venoocclusive Disease (VOD)

Two incidences of VOD were noted in allogeneic BMT. They presented with gallbladder wall edema, hyperammonemia, and bilateral pleural effusion. Ultrasound in both cases showed loss of phasic variation in hepatic vein on spectral waveform. Biopsy was done in one patient to confirm the diagnosis while the other one was clinical diagnosis. No incidences of VOD were noted in autologous BMT.

#### 3.1.4. Acute Graft versus Host Disease (Acute GVHD)

Four incidences of GVHD were noted in allogeneic BMT. Two patients have skin GVHD, one was grade 1 and the other was grade 2. Two were hepatic acute GVHD grade 2/3 with elevated SGPT, bilirubin, and LDH.

#### 3.1.5. Periengraftment Syndrome

Five incidences (10%) (one allogeneic and four autologous) were noted. Common symptoms were weight gain, low grade fever, and electrolyte imbalances, which were treated with short courses of steroids.

#### 3.1.6. Gastrointestinal Complications

These were twelve incidences of grade 2 and grade 3 diarrhoea. There were three incidences of oral and/or anal bleeding.

#### 3.1.7. Renal Complications

Three incidences of acute renal failure (2 autologous and 1 allogeneic) were noted with rise in blood urea nitrogen and creatinine. One incidence of allergic acute kidney injury was noted.

#### 3.1.8. Pulmonary Complications

Two incidences of restrictive lung disease were noted with reduced DLCO and were probably related to previous chemotherapy exposure.

#### 3.1.9. Neurological Toxicity

This was one incidence of peripheral motor and sensory neuropathy.

### 3.2. Late Complications at 100 Days

#### 3.2.1. Chronic GVHD

Two incidences of chronic GVHD (13%) were noted; both were hepatic (limited grade).

#### 3.2.2. Infections

Three incidences of bacterial infections were noted. One incidence was bilateral fungal lung infection and was treated with oral voriconazole. One incidence of Herpes zoster was noted.

### 3.3. Engraftment (Table 7)

By definition, WBC engraftment is absolute neutrophil count >500 for three consecutive days and the platelet engraftment is the platelet count >20,000 for three consecutive days without any external transfusion support [18]. The median engraftment for our autologous BMT was 15 days with range of 10–35 days. The median engraftment days for our allogeneic BMT are 14 days with range of 9–34 days. One allogeneic BMT patient had delayed engraftment because of graft rejection.

### 3.4. Duration of Hospital Stay after Transplant (Table 7)

Average duration of hospital stay after autologous BMT is 18 days with a range of 10–80 days. Average duration of hospital stay after allogeneic BMT is 20 days with a range of 14–70 days.

### 3.5. Mortality, Survival, and Disease Status Statistics (Tables 7 and 8)

Fourteen (40%) of the autologous transplant patients and six (40%) of our allogeneic patients died in the course of follow-up. All of them survived during the first month of posttransplant period. Mortality at 100 days after transplant period also known as transplant related mortality (TRM) in autologous patients was very low with 3% (1/35) while, in allogeneic patients, it was 20% (3/15). Major causes of transplant related mortality were related to bleeding, infections, and acute GVHD. Mortality 1 year after transplant period in our autologous patients was 20% (7/35) and 40% (6/15) in our allogeneic patients. Sixteen (46%) out of our 35 autologous and seven (47%) out of 15 allogeneic patients are in complete remission. 2/35 (6%) autologous HSCT patients have partial remission of the disease while none of the allogeneic transplant patients are in partial remission phase now. 12 (34%) of the autologous transplant patients had disease relapse out of whom nine patients died during the course of follow-up and three patients are alive under some sort of second line management.

### 3.6. Diagnosis Based Mortality and Survival till April 2014

**Figure 3:**
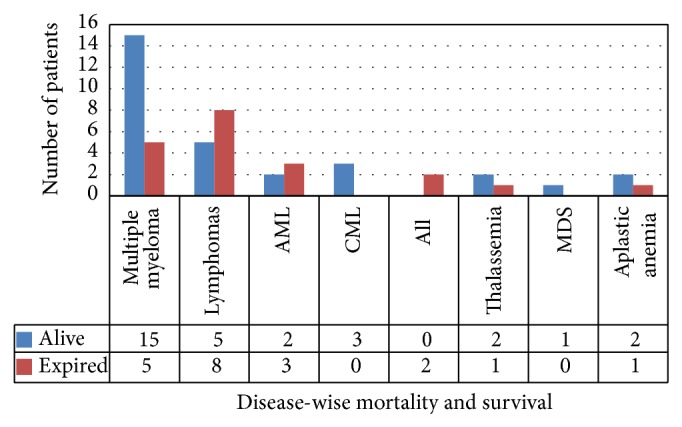


Figure 3 shows the alive and dead patients in various indications of transplants being performed.

**Figure 4:**
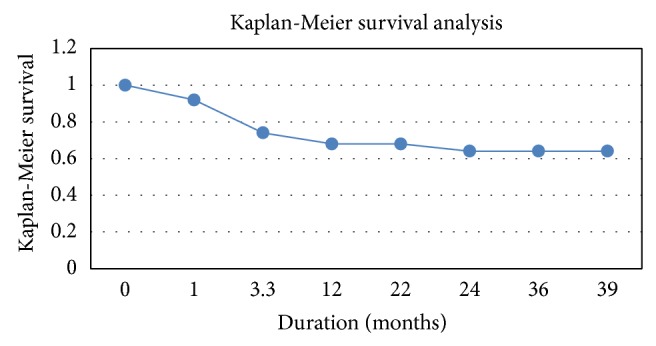
The figure shows Kaplan-Meier survival analysis.

Figure 4 shows number of patients alive at particular posttransplant follow-up period. All of our transplant patients survived during the first month after transplant. Transplant related mortality was four (3 allogeneic and 1 autologous). Major downfall in this graph comes between 100 days and 1 year after transplant period. The major causes of death in this period were neutropenic complications like sepsis, disease relapse, hemorrhagic complications, and GVHD. Our last transplant patient has been followed up for minimum of 22 months, so we can say that all of our patients have at least 22 months of follow-up. 34/50 (68%) patients survived during the first 22 months after transplant period. Number of patients alive at the median follow-up period was 32/50 (66%). The graph plateaus at 1 year of median follow-up after transplant. Two of our autologous patients died because of disease relapse after 39 months in the course of follow-up period.

## 4. Discussion

Apollo Hospital, Gandhinagar, is one of the major bone marrow transplant centres of India. The center is a registered transplant center of CIBMTR (Center for International Blood and Marrow Transplant Registry), USA. This hematopoietic stem-cell transplant data will be the first published data from Western India. The bone marrow/stem-cell transplant center at Apollo Hospital, Gandhinagar, is a new set-up compared to some other set-ups in India and across the world. The first successful autologous transplant was performed on July 5, 2007, in a six-year-old child with Hodgkin lymphoma and the first allogeneic transplant was performed on June 6, 2008, on a thirty-eight-year-old male with AML relapse. Within 5 years span, a total of 50 patients were treated with stem-cell transplants at Apollo Hospital, Gandhinagar. Majority of our HSC collections were done from peripheral blood after G-CSF mobilization. We have not done cord blood transplants, haploidentical donor transplants, or marrow unrelated donor (MUD) transplants yet. We have followed all patients for at least 22 months with median follow-up of 39 months, which is comparable to many national and international studies. Although number of transplants performed is not big enough and data is for shorter duration, still the data is comparable with some national and international published data.

Mean duration of engraftment in our patients was 17 days for autologous BMT and 16 days for allogeneic BMT which is comparable to other standard international data [18]. Only one allogeneic BMT patient had delayed engraftment due to graft failure. Major risk factors of graft failure are disparity between recipient and donor within the major histocompatibility complex (MHC) [19]. We have only used myeloablative conditioning regimen in our transplant patients. One western study shows that the graft failure in myeloablative conditioning regimens is 1/34 compared to 6/24 in nonmyeloablative regimen with *P* = 0.02 [20].

Table 9 shows comparison of various parameters between Apollo Hospital, Gandhinagar, National Cancer Research Institute, Kolkata, Christian Medical College, Vellore, and western studies. Survival in patients of Apollo Hospital is parallel to most of the Indian published reports and many of the western countries.

Health Resources and Services Administration (HRSA), US Department of Health and Human Services, USA, publish their survival analysis data every year at 100 days, 1 year, and 3 years after transplant period [21]. Comparison of survival statistics are analogous to HRSA data from the United States of America but as the numbers of transplants in some of the categories are not sufficient, comparison can be misleading. There are some areas in our efforts, where our Apollo CBCC stem-cell transplant unit is behind some western studies.

For example, infection rates are comparable to other Indian centres but much higher than western studies, which report bacterial infection rate of 5%, viral infection rate of 7%, and fungal infection rate of 12% [8]. The possible risk factors of infections in our patients are aggressive myeloablative conditioning regimens at our center leading to prolonged neutropenia during preengraftment period and possible environmental factors in India [22]. Early posttransplant infection rates are higher in our allogeneic BMT patients probably because we use aggressive myeloablative conditioning regimens which produce very severe and prolonged neutropenia during preengraftment period. One western study demonstrates that, before neutrophil engraftment, the nonmyeloablative cohort had a 53% lower rate of bacterial infections, whereas after engraftment the density of bacterial infections was similar in myeloablative and nonmyeloablative groups. It also shows that, in the first month, both invasive fungal infections and viral infections were twofold less frequent in nonmyeloablative patients [23]. Incidence of viral infections is low (2 CMV and 1 HSV) as compared to two other published data of Indian centres. We do pretransplant evaluation for HIV, hepatitides B and C, and* Cytomegalovirus* (CMV). We also monitor CMV viral load in posttransplant period in all of our allogeneic HSCT patients. Other viruses like Epstein Barr virus and Herpes simplex virus are not monitored regularly.

Major complications during the transplant were GVHD, infections, mucositis, venoocclusive disease, disease related complications, and chemotherapy induced complications and complications related to other comorbidities. One of our myeloma patients required second transplant after the first one because of disease relapse.

Mortality rates are comparable to other Indian and western studies. Major causes of mortality in our patients were infections and disease relapse/progression. Transplant related mortality was found in 8% of our patients. By definition, transplant related mortality (TRM) means deaths occurring during the first 100 days after transplant due to complications of the transplant [24]. Majority of deaths occurred in the first year of posttransplant period. Common causes of non-TRM mortality in the first year after transplant period in our patients were disease relapse (45%), haemorrhagic complications (10%), GVHD (10%), and sepsis (10%).

Overall survival of our autologous and allogeneic HSCT transplant patients was 65.7% and 57.1%, respectively. 46% of our autologous and 47% of our allogeneic patients are in complete remission phase after a median follow-up of 39 months which is comparable to many national and international transplant centres as shown in Table 9. By definition, complete remission means disappearance of all signs of cancer in response to treatment and does not always mean that the cancer has been cured. It is also called complete response [25].

In a developing country like India, there are very few centres, which perform regular HSCT due to various reasons like lack of infrastructure and expertise and lack of knowledge of safety, efficacy, and cost of the procedure both in general population and in medical fraternity. This study will help in sharing its outcomes with other hematology/oncology practitioners and will encourage other centres to start performing stem-cell transplantations or refer eligible patients for this important treatment option available.

## 5. Conclusion

With these encouraging results at our center, we can conclude that our data is comparable to national and international hematopoietic stem-cell transplantation centres in terms of complications, outcomes of treatment, and cost effectiveness. These results provide evidence that the stem-cell transplant, which is a recommended treatment option in various diseases, is possible in a nonuniversity hospital of developing country with excellent safety profile. We will continue to provide our services in the future and try to take them to the next level in terms of application of haplotransplantation, marrow unrelated donor transplantations, and cord blood as the stem-cell source. We will also try to make transplantation possible to some rare indications, nonaffording patients, reduce the complications, and improve the outcomes of the HSCT at our center.

## Figures and Tables

**Table 1:** Baseline patient characteristics and transplant data of our autologous and allogeneic patients.

Characteristics	Autologous	Allogeneic
(1) Total numbers (*N*)	*N* = 35	*N* = 15

(2) Age (median)	50 years	21 years

(3) Gender (M : F)	28 : 7	13 : 2

(4) Indications	Multiple myeloma: 20 Non-Hodgkin lymphoma: 7 Hodgkin lymphoma: 5 Acute myeloid leukemia: 3	Chronic myeloid leukemia: 3 Aplastic anemia: 3 Thalassemia major: 3 Acute lymphoblastic leukemia: 2 Acute myeloid leukemia: 2 Myelodysplastic syndrome: 1 Non-Hodgkin lymphoma: 1

(5) Stem-cell source	All patients from peripheral blood	Peripheral blood: 11 Bone marrow: 4

(6) Stem-cell dose (median cell dose)	2.56^∧^10^6^ cells	5.15^∧^10^6^ cells

(7) Donor type	Not applicable	All siblings

(8) HLA matching	Not applicable	6/6 matched: *N* = 14 4/6 matched: *N* = 1

**Table 2:** 

Conditioning regimens	Indications	Protocol
Autologous HSCT		
BEAM regimen	Hodgkin's lymphoma, non-Hodgkin's lymphoma	Day −6: Carmustine (BCNU) (300 mg/m^2^) Days −5, −4, −3, −2: Etoposide (200 mg/m^2^) immediately after Etoposide infusion Cytarabine (ara-c) (400 mg/m^2^) Day −1: Melphalan (140 mg/m^2^/dose) Day 0: stem-cell transplant
Melphalan regimen	Multiple myeloma	Day −1: Melphalan 200 mg/m^2^ or Melphalan 140 mg/m^2^ Day 0: stem-cell transplant
FluMel	NHL Follicular lymphoma	Fludarabine 30 mg/m^2^ for 4 days, on days −7 to −4 Melphalan 70 mg/m^2^ on days −3, −2
Allogenic HSCT		
BuCy regimen	AML, CML, ALL	Days −7, −6, −5, −4: Busulfan: 3.2 mg/kg/day IV Days −3, −2: Cyclophosphamide 60 mg/kg/day Day 0: stem-cell transplant
Cyclophosphamide + ATG	Aplastic anemia	Cyclophosphamide 200 mg/kg on days −5, −4, −3, −2; ATG 90 mg/kg on days −5, −4, −3
Cyclophosphamide + thiotepa + oral busulfan	Thalassemia	Days −10 to −2: oral Busulfan 4 mg/kg/day for 4 days; Thiotepa 10 mg/kg once; Cyclophosphamide 200 mg/kg over 4 days

**Table 3:** Graft versus host disease prophylaxis.

Day	Prophylaxis regimen
−1	Cyclosporine 2.5 mg/kg/dose twice daily

0	Peripheral stem-cell transplant

0	Cyclosporine 1.5 mg/kg/dose twice daily; continued till day +90 and then gradually taper the dose

+1, +3, +6, +11	Methotrexate 15 mg/m^2^ on D +1, 10 mg/m^2^ on rest of days [6]

+2, +4, +7, +12	Two doses of leucovorin 15 mg/kg/every six hours starting 24 hours after methotrexate

**Table 4:** Our antimicrobial prophylaxis for autologous BMT is as follows.

Antibiotics	Dose	Duration
Trimethoprim-sulfamethoxazole single (double strength for adults) strength	1 tab. once daily	Stops on day −2 and is restarted after day 28 or engraftment and is continued for 6 months

Tab. of valacyclovir	500 mg twice daily	Starts on day −2 till discharge

Tab. of fluconazole	200 mg twice daily	Starts on day −7 till discharge

Tab. of levofloxacin	500 mg once daily	Starts on day −7 till discharge

**Table 5:** Our antimicrobial prophylaxis for allogeneic BMT is as follows.

Antibiotics	Dose	Duration/special comments
Tab. of levofloxacin	500 mg once daily	Day −3 till discharge

Capsule fluconazole	200 mg twice daily	From day −3 to day +75 or till patient is immunocompromised due to likely GVHD management as fungal prophylaxis or amphotericin B 0.5 to 1 mg/kg once daily or on alternate days after ANC becomes less than 200; antifungal prophylaxis stops after neutrophil engraftment

Tab. of valacyclovir	1000 mg once daily	From day −3 to day +30 or longer in case of GVHD as Varicella zoster virus and Herpes simplex virus prophylaxis

Trimethoprim/sulfamethoxazole	10 mg/kg/day	Double strength daily from day −8 till day −2; it is restarted twice weekly as soon as engraftment is achieved

Tab. of penicillin V	250 mg twice weekly	From day +28 for prophylaxis against encapsulated organisms or tab. of amoxicillin-clavulanate for *Pneumocystis carinii *prophylaxis

Febrile neutropenia episodes were managed as per standard protocols.

**Table 6:** 

Type of complication	All grades numbers = *N*	Grade 3 or 4 numbers = *N*
(1) Oral mucositis	Allogeneic: *N* = 4 Autologous: *N* = 5	Allogeneic: *N* = 2 (all grade 3, no grade 4) Autologous: *N* = 2 (all grade 3, no grade 4)

(2) Venoocclusive disease	Grades not applicable Allogeneic: *N* = 2 Autologous: *N* = 0	Grades not applicable

(3) Acute GVHD	Allogeneic: *N* = 4 Autologous: not applicable	Allogeneic: *N* = 2 (both grade 3 hepatic acute GVHD; no grade 4 GVHD noted) Autologous: not applicable

(4) Periengraftment syndrome	Grades not applicable Allogeneic: *N* = 1 Autologous: *N* = 4	Grades not applicable

(5) Diarrhoea	*N* = 12 in autologous and allogeneic	*N* = 7 in autologous and allogeneic

**Table 7:** Outcome parameters of our autologous and allogeneic patients till April 2014 after a median follow-up of 39 months.

Outcome data	Autologous	Allogeneic
(1) Median engraftment day (Range)	15 days (10–35 days)	14 days (9–34 days)

(2) Median posttransplant hospital stay (range)	18 days (10–80 days)	20 days (14–70 days)

(3) Mortality rate	30-day mortality: *N* = 0 100-day mortality: *N* = 1 1-year mortality: *N* = 7 3-year mortality: *N* = 12 39-month (median follow-up) mortality: *N* = 12 Current mortality: *N* = 14	30-day mortality: *N* = 0 100-day mortality: *N* = 3 1-year mortality: *N* = 6 3-year mortality: *N* = 6 39-month (median follow-up) mortality: *N* = 6 Current mortality: *N* = 6

(4) Current disease status	Complete remission: *N* = 16 Partial remission: *N* = 2 Relapse but alive: *N* = 3	Complete remission: *N* = 7 Partial remission: *N* = 0 Relapse but alive: *N* = 2

(5) OS at median (39 months) follow-up	65.7% (*N* = 23)	57.1% (*N* = 8)

**Table 8:** Outcomes of our autologous and allogeneic HSCT patients.

Outcomes of our transplant patients	Autologous HSCT (*n* = 35)		Allogeneic HSCT (*n* = 15)	
Complete remission	46% (16/35)		47% (7/15)	

Partial remission	6% (2/35)		0% (0/15)	

Relapse but alive	8% (3/35)		13% (2/15)	

Transplant related mortality (TRM)	3% (1/35)		20% (3/15)	

Non-TRM	37% (13/35)	Causes	20% (3/15)	Causes
		Disease relapse 9		
		Chronic GVHD 1		ARDS with multiorgan failure 1
		Sepsis with multiorgan failure 1		
		Haemorrhagic brain infarction 1		Haemorrhagic cystitis 1
		Chronic GVHD 1		Resistant chronic GVHD 1

**Table 9:** 

	Apollo Hospital International Limited, Gandhinagar (*N* = 50)	Christian Medical College, Vellore (*N* = 221) [7]	National Cancer Research Institute, Kolkata (*N* = 22) [8]	Western studies
Bacterial infections	50%	34.9%	52%	5%

Viral infections	4%	42.9%	24%	7%

Fungal	14%	15.9%	12%	16%

Blood culture positivity	22%	53.8%	50%	12.5% [9]

Incidence of gram negative infection	22%	80%	80%	11.2% [9]

Graft versus host disease	Grade 1 skin GVHD in 6.7% (1/15), grade 2 skin GVHD in 6.7%, and acute hepatic GVHD grade 2-3 in 13.3% (2/15)	17% grade 3 and grade 4	Skin GVHD grade II in 18.2%, grade I GVHD of liver in 13.6%, and grades II-III gut GVHD in 9%	Data not available

Mortality rate	No mortality for the first month; 100 day mortality 8%; overall mortality 40% with the median follow-up of 39 months	Overall mortality is approximately 28%	Overall mortality was 13.7% at the median follow-up of 4.6 years	Mortality rate for allogeneic transplant is approximately 30% and for autologous transplant it is approximately 10% after 3 years of follow-up.(USA and Canada) [10]

Long term survival	Overall survival for thalassemia patients, autologous HSCT patients, and allogeneic HSCT patients was 66%, 65.7, and 57.1%, respectively, with median follow-up of 39 months	Overall survival 72.3 ± 3.1% of 218 patients of thalassemia at median follow-up of 5 years	Overall survival 86.3%; disease-free survival 68.2% seen at median follow-up of 22 patients for 4.6 years	50–70% in chronic leukaemias and 80–90% with aplastic anaemias (USA and Canada) [11]
